# Supplementary material for: Mechanics of Drosophila wing deployment
Source: Nat Commun. 2024 Dec 11;15:10577. doi: 10.1038/s41467-024-54527-0 (PMC11634967; doi:10.1038/s41467-024-54527-0)
Supplement: Supplementary file 1 — Supplementary Information [file 41467_2024_54527_MOESM1_ESM.pdf]

Supplementary Information
Mechanics of *Drosophila* wing deployment

Simon Hadjaje[1], Ignacio Andrade-Silva[1,2], Marie-Julie Dalbe[3],
Raphaël Clément[4*], Joel Marthelot[1*]

[1]Aix-Marseille University, CNRS, IUSTI & Turing Centre for Living Systems (CENTURI), Marseille, France

[2]Departamento de Física, Facultad de Ciencias Físicas y Matemáticas,Universidad de Chile, Santiago, Chile

[3]Aix-Marseille University, CNRS, Centrale Mediterranée, IRPHE, Marseille, France

[4]Aix-Marseille University, CNRS, IBDM & Turing Centre for Living Systems (CENTURI), Marseille, France

1 Macroscopic origami folding and vein network

Newly eclosed fly exhibits highly folded wings. Supplementary Fig. 1a shows a folded fly wing (top: dorsal side; bottom: ventral side) with the veins used as landmarks throughout this study indicated in colors (see Fig. 2 of the manuscript). These stereotypical folds, which occur along the longitudinal veins but also in the perpendicular direction ("marginal fold" at the proximal end of the wing [1]), unfold within minutes with equally stereotyped dynamics. Supplementary Fig. 1b shows an adult wing and the associated vein network.

Wing deployment is a dual morphing mechanism that involves unfolding macroscopic folds and additional isotropic tissue stretching. In Supplementary Fig. 1c we quantify the macroscopic deployment $\Lambda = L/L_F$ in time (solid dark green line), with L the length of the straight segment connecting the two extremities of the longitudinal vein shown in green in Supplementary Fig. 1a-b, and L_F the corresponding length in the folded state. We compare this dynamics of unfolding with the dynamics of the tissue stretch $\lambda = l/l_F$ (shown as the dashed green curve), where l is the arclength of the same green vein, and l_F the corresponding folded arclength. We note that the two mechanisms appear to be simultaneous.

2 Quantification of curvature during deployment

Wing deployment is characterized by a complex change of curvature. The wing is initially flat when folded, then curves downwards as soon as it begins to unfold. It then returns to a flat, expanded plate, before reversing its curvature slightly at the very end of deployment in the transverse direction. To quantify this change in curvature, we track the edge of the wing on side- and top-view recordings (see Supplementary Fig. 2a-b). The side-view perspective gives the wing profile represented by a dotted line in the inset of Supplementary Fig. 2a, and the

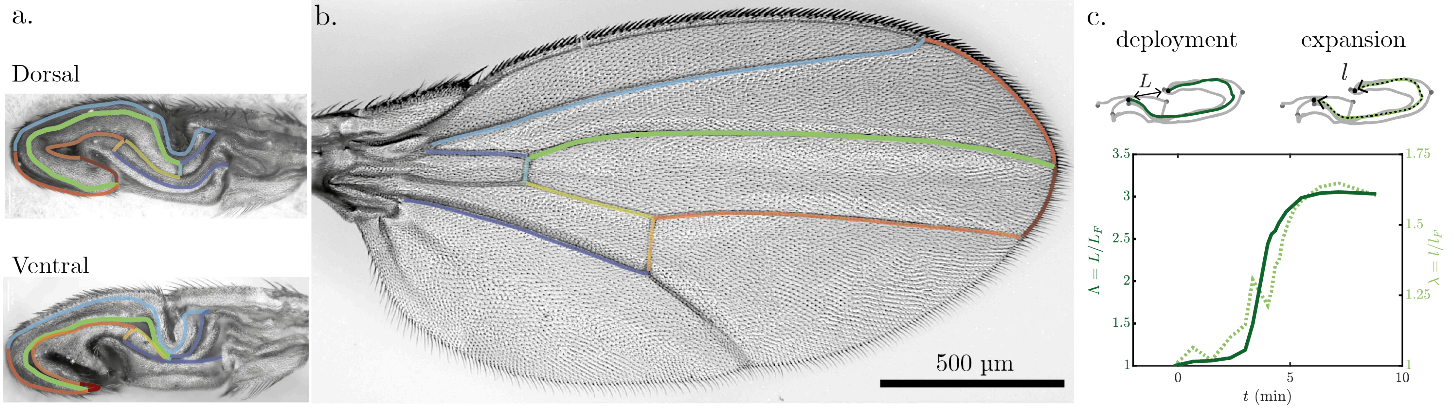

Supplementary Figure 1: **Folded and deployed wings with their corresponding vein network.** **(a)** Dorsal and ventral side of a folded wing. Most of the veins follow the macroscopic folds and are located either on a mountain or in a valley fold. **(b)** Adult wing with its anterior edge at the top. The highlighted veins in a-b are the one that can also be visualized on the micro-CT scans. **(c)** Wing deployment Λ and stretch λ as a function of time during the deployment process.

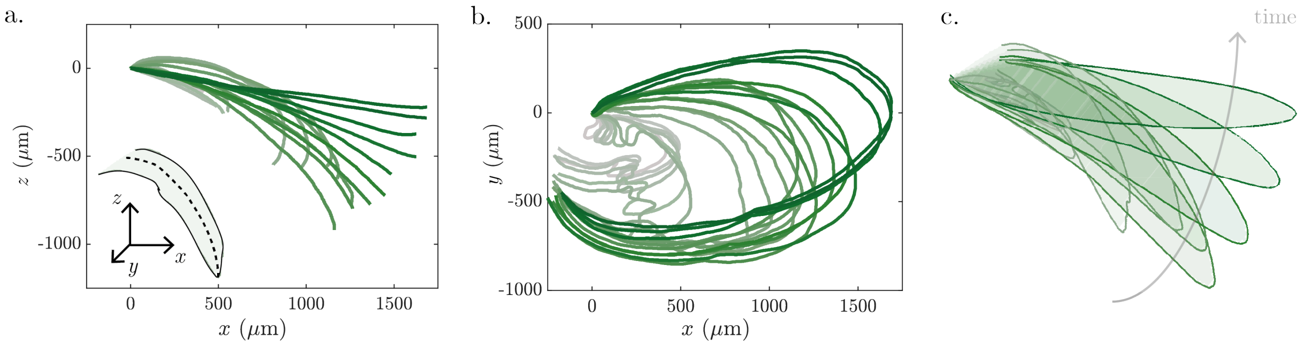

Supplementary Figure 2: **Curvature during deployment in wild-type wings.** **(a)** Side-view and **(b)** top-view profiles of the marginal vein (i.e. the vein that forms the contour of the wing). Time is color-coded from light to dark green. (c) 3D reconstruction of a wing during deployment.

top-view gives the marginal wing contour (solid line in the inset). The different colors of the wing profile in Supplementary Fig. 2a correspond to different times during deployment (from 4:00 in light gray to 8:30 in dark green) and show that the wing curves downwards during deployment. Supplementary Fig. 2b shows the top-view of the wing silhouette during deployment from a compact folded structure (light gray) to a fully deployed wing blade (dark green). The corresponding schematic 3D reconstruction of the wing during deployment is shown in Supplementary Fig. 2c.

Other changes in curvature during wing deployment can also be observed in mutants flies such as *Curly* mutants, which are widely used to track and identify mutations. When working with genes that are not associated with immediately visible phenototypes, these mutations are often combined with the *Curly* mutation to highlight the mutation of interest through macroscopic observation. A few studies have attempted to elucidate the physical mechanism leading to the *Curly* phenotype, but a mechanical description remains missing. Using transmission

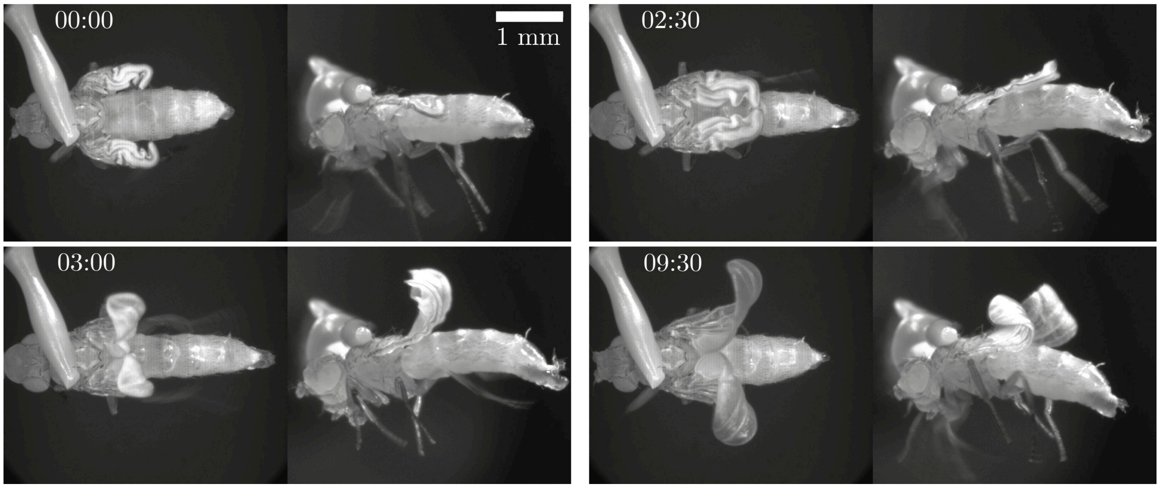

Supplementary Figure 3: ***Curly* mutant wing deployment at the organ level.** Snapshots of *Curly* wing from a top and lateral view during deployment.

electron microscopy, Hurd et al. [2] observed that the wings of *Curly* mutants occasionally show abnormal pairing between the dorsal and ventral cuticle. One hypothesis is that these anomalies lead to a reduction in the surface area of the dorsal wing, causing the wing to curve upwards.

We characterize the kinematics of *Curly* flies during deployment (see Supplementary Fig. 3). Although the initial macroscopic folding pattern of the wing is identical to that of the wild-type, the wings unfold out of plane when deployment begins. In adults, the wings are characterized by a permanent upward curvature. Adult *Curly* flies are therefore unable to fly, and move around in small hops.

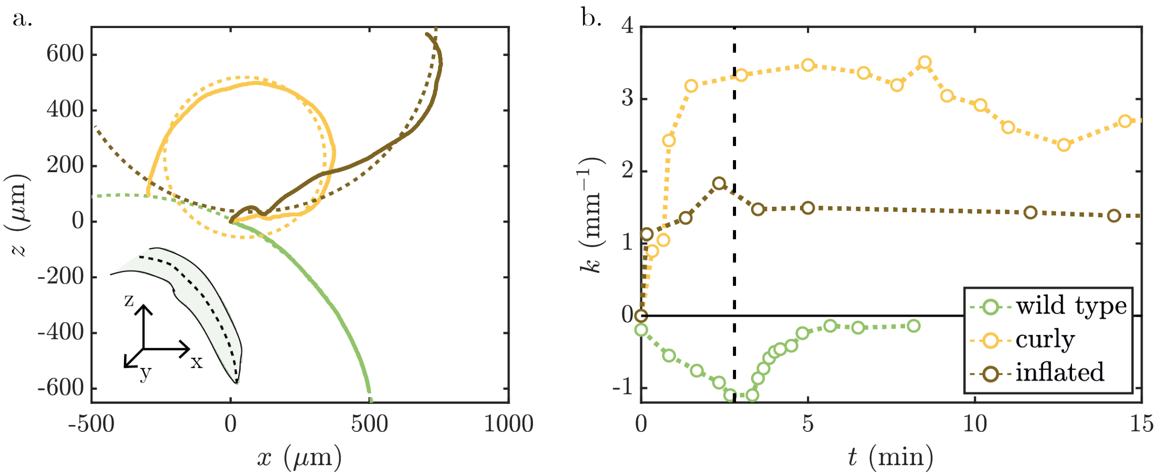

Supplementary Figure 4: **Wing curvature in wild type, *Curly* and artificially inflated wings. (a)** Shapes of wild-type, *Curly* and inflated wings during the deployment process. **(b)** Evolution of the average side-view curvature of the wing k as the wing deploy. The vertical dashed line corresponds to the profile in (a).

We now quantitatively evaluate the difference in curvature dynamics between wild type fly, *Curly* mutant and artificial inflation of wild-type fly. We fit the side-view with a circle to calculate the average curvature

$k = 1/R$ (with R the radius of the fitted circle). Supplementary Fig. 4a shows the side-view of the marginal vein 3 minutes after the onset of wing deployment for a wild-type (green), *Curly* (yellow) and artificially inflated fly (brown). As shown in Supplementary Fig. 4b, the wild-type wing is initially flat when folded ($k = 0$), and curves downwards as soon as it begins to unfold ($k < 0$). It then returns to a flat, expanded plate ($k = 0$). For both *Curly* mutants and artificial inflation, the wing immediately curves upwards ($k > 0$) at the beginning of deployment, and reaches a plateau: the adult fly has a positive curvature. While artificially inflated wings qualitatively resemble the phenotype observe in *Curly* wings, they are quantitatively different, *Curly* wings being 2 to 3 times more curved than artificially inflated fly wings.

3 Geometrical parameters measured from micro-CT scan

We use ImageJ to extract the geometrical parameters of the folded wing from micro-CT scans. As sketched in Fig. 1b of the manuscript, the folded wing is composed of two plates of thickness e connected through pillars of diameter d, height h and organized in a hexagonal lattice of spacing a. We apply thresholding, ultimate points and Voronoi tesselation on high-resolution scans (see Fig. 1b(iii) of the manuscript, 1 voxel = 0.32 μm) to measure the diameter of the pillars $d \approx 3.3\,\mu$m and the distance between them $a \approx 6.7\,\mu$m. We take advantage of lower-magnification scans (see Supplementary Fig. 5a, 1 voxel = 0.8 μm) to measure the plates thickness e and the gap between them h. We obtain a first binary mask by applying a threshold on the upper and lower plates (see Supplementary Fig. 5b). The open surface area of this mask thus gives $S_0 = 2el$ where l is the arclength of the section. We then apply a closing process to obtain the full silhouette of the section (see Supplementary Fig. 5c). We obtain l by performing a skeleton process on this last mask and get the average plate thickness $e = S_0/(2l) \approx 6.5\,\mu$m. The close surface area of the mask also yields the surface $S = (2e + h)l$, which allows calculating the average gap $h = (S - S_0)/l \approx 7.5\,\mu$m. The total thickness of the wing (composed of the plates thickness and the gap in between) while the wing is deploying is obtained with two-photon imaging (see Supplementary Fig. 5d and Supplementary Movie 7).

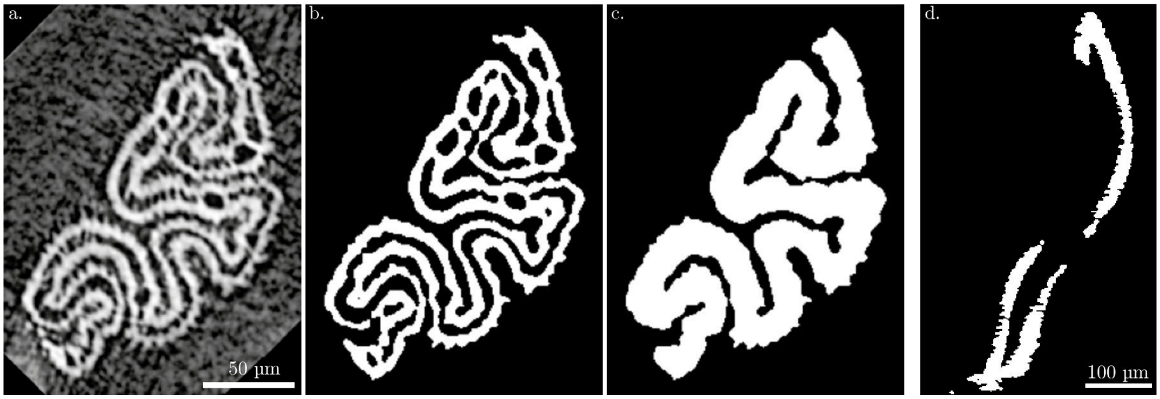

Supplementary Figure 5: **micro-CT and two-photon post-processing. (a-c)** micro-CT: (a) Original cross-section normal to the proximo-distal axis of a folded wing. **(b)** Threshold highlighting the top and bottom plates. **(c)** A closing process yields the full silhouette of the section. **(d)** Two-photon: Cross section of a deploying wing.

4 Volcano-like shape of the apical cell surface

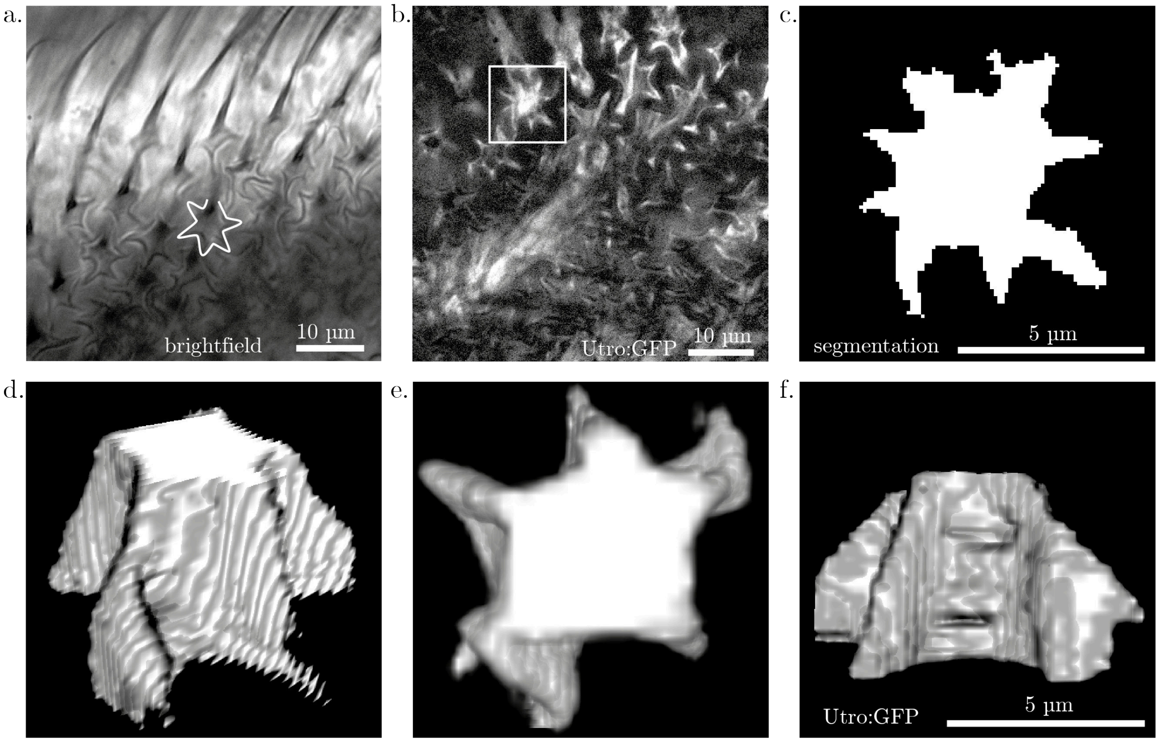

Supplementary Figure 6: **Volcano-like shape of the apical cell surface. (a)** Brightfield micrographs showing the contour of epithelial cells (highlighted in white) and wing hairs (dark shadows) in folded wings. **(b)** Utrophin:GFP signal epithelial cells in folded wings. **(c)** Segmentation of the cell inside the white square in (b). **(d-f)** 3D reconstruction of the apical surface of single cell.

Before deployment, the cuticle is wrinkled, and each wrinkle corresponds to a cell whose apical surface has a particular 3D arrangement. Bright-field microscopy images already reveal the buckled contours of the cells in the folded wings (see Supplementary Fig. 6a). The wing hairs appear as black dots in the center of each cell. The outline of a single cell is highlighted in white: it has the shape of a star, with each branch intertwined with neighboring cells. Utrophin:GFP folded wings, shown in Supplementary Fig. 6b, allow the 3D shape of individual cells to be segmented. We crop a region of interest that captures a cell and threshold to obtain only the shape of the cell (see Supplementary Fig. 6c). We perform this process on the entire stack covering the cell and reconstruct the 3D shape of the cell by re-stacking the segmented images shown in Supplementary Fig. 6d-f. In folded wings, the apical surface of the cells is shaped like a volcano or truncated cone, with straight ridges on the sides. This shape is isometric to a plane and allows the cuticle to unwrinkle without stretching as the wing surface expands.

5 Isotropic in-plane properties of the tissue

The potential anisotropy of the tissue cannot be measured directly by tensile methods due to the initial macroscopic folding of the wing, so we have to rely on indirect observations. We assume that the elastic response of the wing

is isotropic in-plane at the mesoscopic scale from three observations:

(1) The wing presents a highly regular three-dimensional structure, characterized by volcano-like wrinkles arranged in a hexagonal tiling associated with the hexagonal organization of epithelial cells. To highlight this hexagonal tiling, we perform a Fourier transform of the folded and deployed wing surface based on profilometric measurements of the wing topography (see Supplementary Fig. 7). In both cases, we find a hexagonal organization of the volcano-like wrinkle characterized by 60-degree angles in reciprocal space, with a larger pattern wavelength for the deployed wing. While this hexagonal organization might suggest an anisotropic response of the structure, we observe that the peaks in inverse space remain on a circle in the deployed state, indicating that the extension is isotropic in-plane.

(2) At the macroscopic scale, we characterize the stretching of the veins as the wing expands under an isotropic pressure load. We observe that veins oriented in different directions exhibit the same stretch, indicating that extension is isotropic in the plane (see Fig. 2c of the manuscript).

(3) At the microscopic scale, we have imaged the contour of epithelial cells using spinning-disk confocal microscopy and Ecad:GFP in Fig. 3d of the manuscript. In the intervein region, we observe that while cell surface area increases during expansion under an isotropic pressure load, cell shape remains isotropic hexagonal after expansion.

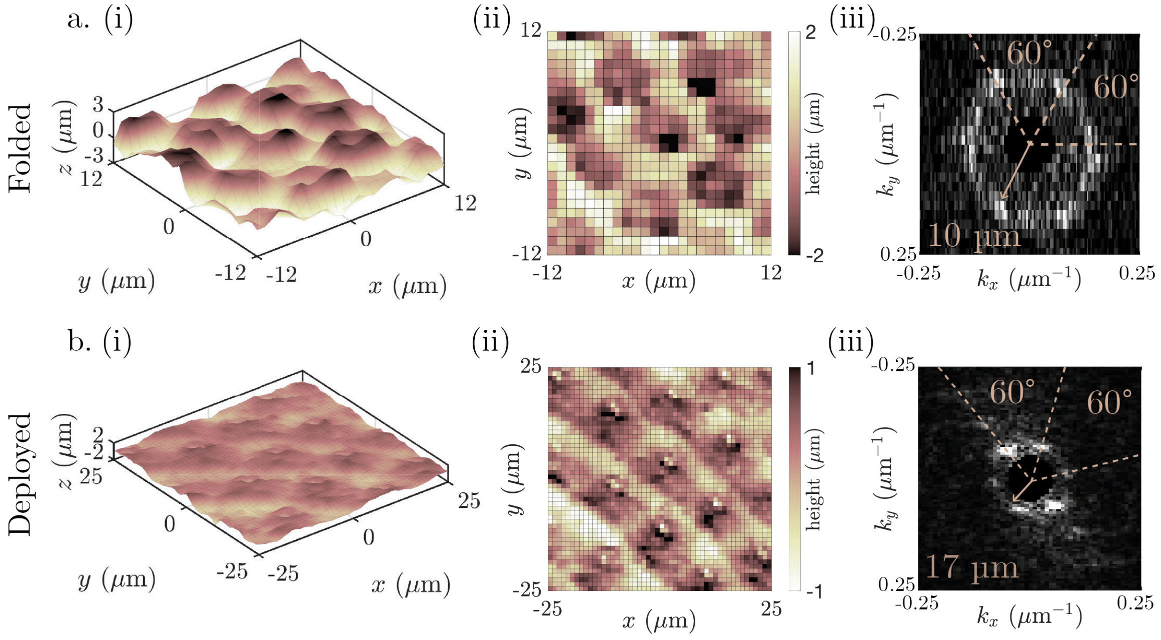

Supplementary Figure 7: **Volcano-like wrinkle hexagonal tiling in folded (a) and deployed (b) wings.** (i-ii) Topography of the wing surface; (iii) Fourier transform of the wing surface topography. Peaks in reciprocal space with angle of $\approx 60^\circ$. Distance from the center gives the inverse of the pattern wavelength.

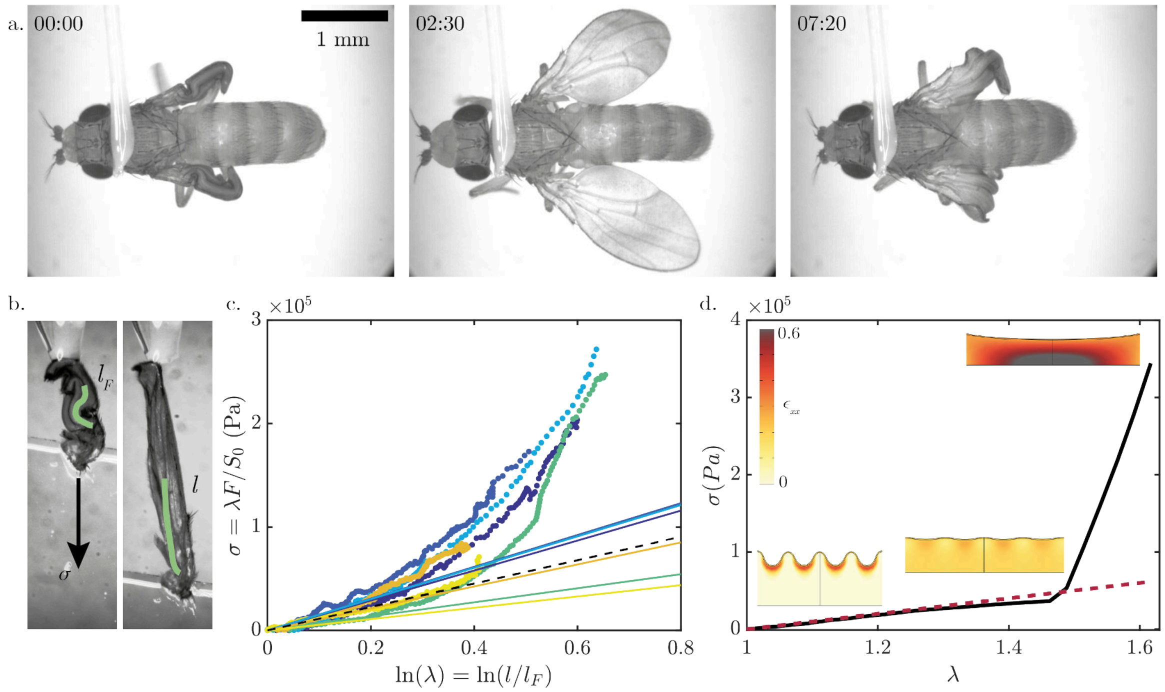

Supplementary Figure 8: **Tensile experiment.** **(a)** Failure of wing expansion in a wild-type fly with elastic recovery (see Supplementary Movie 13). **(b)** Pictures of a tensile test experiment with the folded wing glued at both distal and proximal extremities. The followed vein is underlined in green. **(c)** True stress $\sigma = \lambda F/S_0$ versus true stretch $\ln(\lambda) = \ln(l/l_F)$ for all of the six experiments (data: colored markers; corresponding linear fit: colored solid lines; averaged fit: dashed black line). **(d)** FEM numerical simulation of the stretching of a wrinkled bilayer and computation of the associated stress-stretch. Color code is the strain in the tensile direction ϵ_{xx}. Red dotted line: theoretical prediction of $\sigma - \lambda$ relationship for the substrate alone.

6 Wings mechanical properties measured through tensile tests

Supplementary Fig. 8a shows snapshots of a wild-type fly that begins to expand its wings, but whose internal pressure decreases before the process is complete. We observe that the wings fold back, imperfectly, but with their longitudinal vein folds and marginal folds. This reversibility of folding prompts us to use an elastic description to model the deformation of the structure.

Tensile tests are conducted on dissected folded fly wings to characterize their mechanical properties. A displacement is imposed on the proximal end of the wing, which is attached to a linear stage. The force, F, is measured using a load sensor attached to the distal end of the wing (see pictures of the experiment in Supplementary Fig. 8b). Supplementary Fig. 8c shows the true stress for six experiments (colored markers) as a function of the true strain obtained by direct measurement of the variation in one of the longitudinal veins (shown in Supplementary Fig. 8b) and their associated linear fit at small strains (colored lines), which yield the Young's modulus E. The average linear fit is shown as a dashed black line.

The strain stiffening of a wrinkled bilayer as it unwrinkles is illustrated using 2D FEM numerical simulations (COMSOL Multiphysics). The bilayer consists of a soft 10 μm thick substrate of epithelial cells with a Young's

modulus $E = 100\,\text{kPa}$ covered by a 200 nm thick rigid film of cuticle with a Young's modulus $E_f = 100\,\text{MPa}$. To improve convergence, we consider a hyperelastic Gent model for the substrate with an arbitrarily large value of $J_m = 100$, which has a negligible impact for the values of stretching considered in the simulation. We impose symmetric boundary conditions on the bottom and left sides of the system while we prescribe the displacement on the right side. The top surface is free.

The initial state is obtained by thermally contracting the substrate to form wrinkles (see FEM snapshot, bottom left in Supplementary Fig. 8d). This numerical method is commonly used to study instabilities resulting from differential growth in biological structures [3]. Thermal expansion and biological growth are rigorously kinematically equivalent [4]. To obtain the initial state of the wrinkled cuticle bilayer, we define a coefficient of thermal expansion in the longitudinal direction x of the epithelial cell substrate with a thermal strain $\epsilon_{th} = \alpha_{xx}\Delta T$, where α is the coefficient of thermal expansion. When a finite temperature difference ΔT is applied, the substrate shrinks along the longitudinal direction, so that the film becomes comparatively too long and buckles.

We then impose a displacement on the right boundary condition. Supplementary Fig. 8d shows the normal stress along the right boundary condition as a function of the stretch, λ. The curve shows two distinct regimes: (i) at moderate stretch ($\lambda < 1.5$), stress is low and is dominated by the stretching of the soft substrate, while the unwrinkling of the rigid film has a minimal impact on the overall stiffness of the bilayer. Quantitatively, the effective stretch modulus of the bilayer is similar to the elastic response of the substrate (rod dotted line); (ii) as the wrinkles disappear (intermediate snapshot $\lambda \sim 1.5$), the system becomes stiffer and the mechanical response is dominated by the stretching of the rigid film.

These simulations illustrate the global strain stiffening of the wing observed in the experiments, which results from microscopic unwrinkling of the cuticle, and support the choice of the Gent's hyperelastic model to account for the effective mechanical response of the bilayer forming the wing.

7 Nanoindentation of the cuticle of adult wings

The adult wing is composed of a thin flat layer of sklerotized chitin with a total thickness of approximately $500\,\text{nm}$ in the intervein regions. We perform nanoindentation experiments on fully deployed adult wings. The wings are dissected and placed on a thin layer of superglue coated on a glass plate. We impose loading and unloading force cycles and obtain the $F_i - \delta$ curve shown in the Inset of Supplementary Fig. 9a. We perform multiple indentations on different areas of adult wings on 5 different samples.

In Supplementary Fig. 9a, we plot on a logarithmic scale the elastic discharge in order to exclude plastic effects observed during loading. The curves line up with a slope of 3/2 in agreement with the prediction of the Hertz contact model for a rigid spherical probe indenting a soft, elastic, planar sample:

$$F_i = \frac{4}{3}\frac{E_c}{1-\nu^2}R^{1/2}\delta^{3/2} \tag{1}$$

with E_c the cuticle Young's modulus, $\nu = 0.5$ its Poisson's ratio, and R the radius of the indenter. We invert this expression to extract the Young's modulus E_c of each sample.

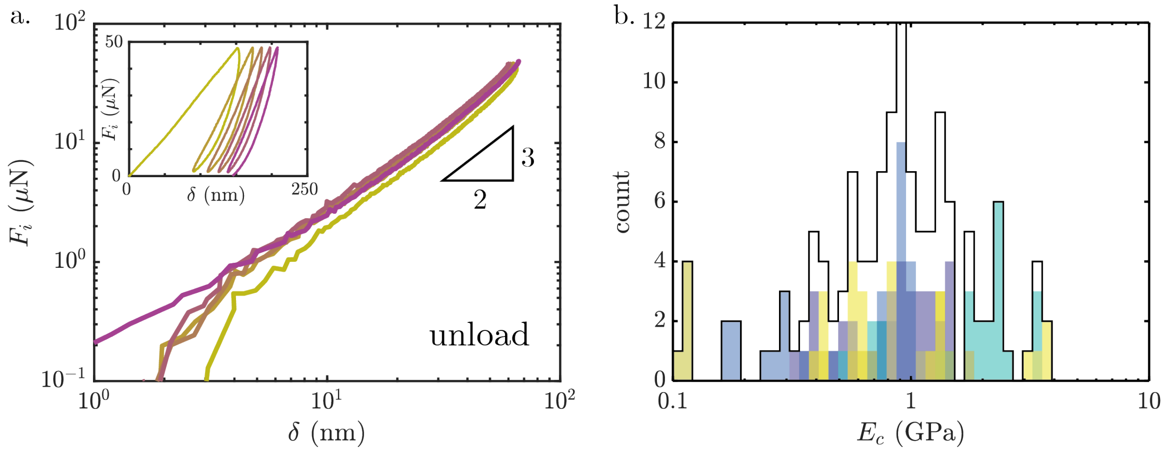

Supplementary Figure 9: **Nanoindentation of adult wing.** **(a)** Force-displacement indentation of an adult wing on a logarithmic scale (unloading phase only). Inset: raw $F_i - \delta$ curve. **(b)** Histogram of Young's modulus E_c of adult wings. Each color corresponds to a different fly (n=5), the black line is the cumulative histogram.

All measurements are shown in Supplementary Fig. 9b, with each color corresponding to a different fly. Different data for a fly correspond to different indentation times and positions. The cumulative histogram is represented by the black contours and shows a peak at $E_c \approx 1$ GPa. The dispersion observed from one wing to the next can be explained by the fact that the cuticle undergoes sclerotization after deployment, which tends to increase cuticle stiffness [5]. The stiffness measured is very consistent with AFM measurements on adult wings in the literature [6].

8 Model parameters

The wing expansion model depends on both mechanical parameters (Young's modulus E, Gent limiting value J_m) and geometrical features (plates thickness e, pillars height h, diameter d, interpillar distance a). The aforementioned parameters are quantified through microscopic characterization and mechanical testing. However, it is of interest to assess the impact of these parameters on model predictions, and the robustness of predictions to small parameter variations.

Supplementary Fig. 10a illustrates that at small strain ($\lambda < 1.5$), the linear elastic law does not differ greatly from a hyperelastic material. A purely linear elastic model predicts a finite pressure at which stretching, in both horizontal and vertical directions, diverges. We therefore opt for a more realistic model that incorporates the effects of large strains and non-linearities (strain-stiffening) in the material [7]. The value of J_m is determined by matching predictions of the stretch in the pillars λ^p (Supplementary Fig. 10b) with experimental observations. The maximum pillar stretch measured *in vivo* is $\lambda^p \sim 4.8$. A value of $J_m = 20$ corresponds well to this limiting value at working pressure $\overline{P} \sim 0.18$. To keep the model simple, we have chosen to use the same parameter J_m for the biaxial expansion of the plates.

Supplementary Figures 10c-d illustrate the influence of geometry on the prediction for the in-plane stretch λ (top graphs) and the stretch in the pillars in λ^p (bottom). We note in particular that more slender pillars (i.e. $\Psi \sim 1$

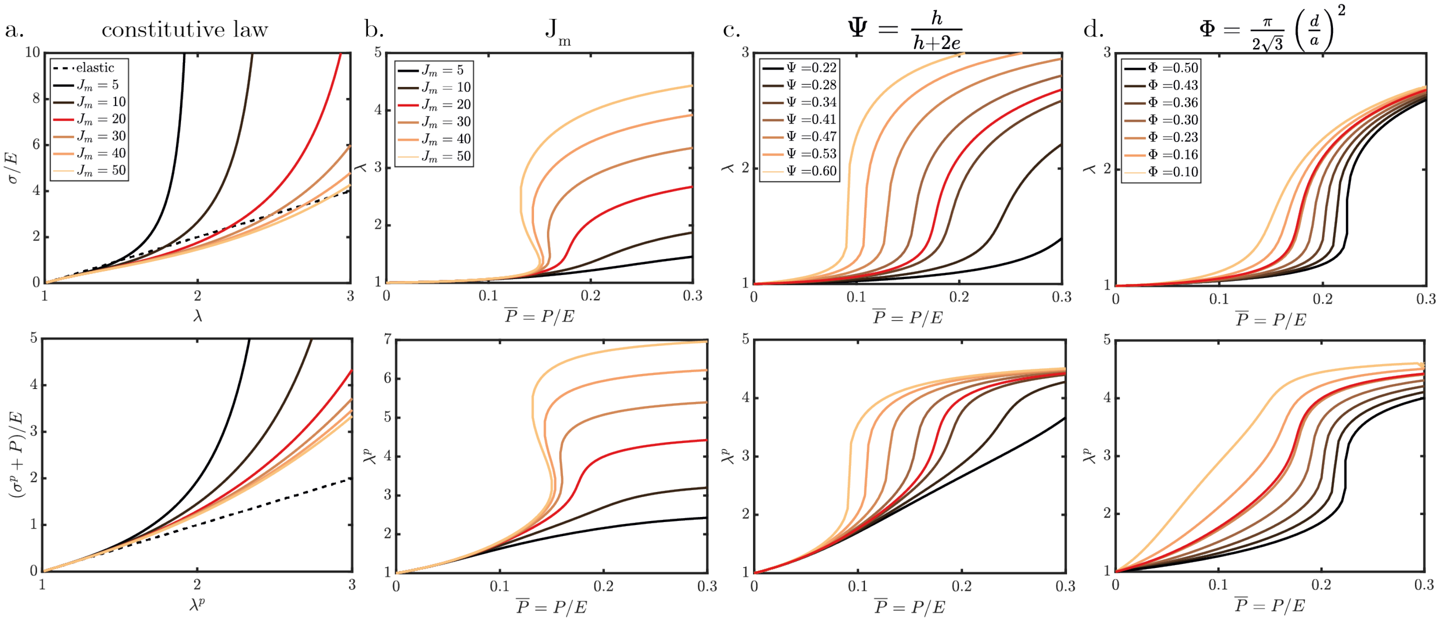

Supplementary Figure 10: **Model and influence of the mechanical and geometric parameters.** **(a)** Elastic vs hyperelastic constitutive law: (top) in-plane normalized stress σ/E as a function of the in-plane stretch λ and (bottom) perpendicular normalized stress $(\sigma^p+P)/E$ as a function of the pillar stretch λ^p for an elastic constitutive law (dotted line) and hyperlelastic Gent models (solid lines, different parameters J_m). **(b-d)** (top) in plane stretch λ and (bottom) perpendicular stretch λ^p versus normalized pressure $\overline{P}$ varying parameters J_m (b), pillars relative height Ψ (c), and in-plane pillars density Φ (d).

or $\Phi \sim 0$ in terms of non-dimensional parameters) result in a shift of the curves towards lower working pressure.

To validate our model, we perform FEM numerical simulations of the inflation of a wing-shaped structure composed of a hyperelastic Gent material ($E = 100\,$kPa, $J_m = 20$). Supplementary Fig. 11a shows simulation predictions of the in-plane stretch λ as a function of the normalized applied pressure $\overline{P}$ (dotted lines) for which we vary the system size (36, 64 and 37 pillars) and pillar arrangement (square and hexagonal lattice, adjusting the distance a between the pillars to keep the pillars density $\Phi = 0.22$ constant, see FEM snapshots Supplementary Fig. 11b). All predicted λ follow the same curve, which shows that (i) the system is sufficiently large for boundary effects to be negligible; and (ii) confirms that pillar organization has no key role in the model, the important parameter being the pillar density Φ. It should be noted that our simple model (shown in black solid line Supplementary Fig. 11a) does not take into account the deformation offset at the connection between the pillars and the plates, (i.e. during inflation, the plates stretch while the pillars shrink in the xy-plane), which is likely to contribute to the discrepancy observed with the FEM.

9 Viscous dissipation probed through nanoindentation experiments

We perform creep tests and loading-unloading cycles in nanoindentation to measure the wing material viscous dissipation. Supplementary Fig. 12a-c show three creep tests for which a constant force F_i is applied (see Supple-

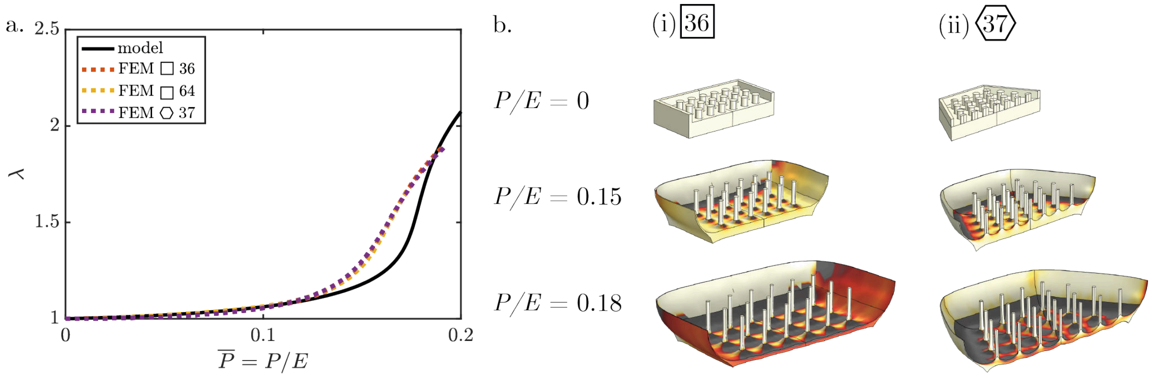

Supplementary Figure 11: **FEM simulations of inflating a hyperelastic wing-shaped structure.** **(a)** Stretch λ as a function of the normalized applied pressure $\overline{P}$: all FEM predictions (dashed lines) varying the size of the system (36, 64 and 37 pillars) and the pillars organization (square and hexagonal lattice) collapse on the same curve. The model is shown as the black solid line. **(b)** FEM snapshots at different pressure of 1/4th of the geometry for (i) 36 pillars organized in a square lattice, and (ii) 37 pillars organized in a hexagonal lattice. Color code: strain in one of the in-plane principal direction.

mentary Fig. 12d for the loading signal). Each curve is fitted by a three elements Maxwell-Jeffrey model (yellow line), yielding to a short timescale $\tau_1 = 1.9 \pm 0.3$ s, a long relaxation time, $\tau_2 = 37 \pm 12$ s and an effective elastic response of the bilayer in indentation $E_i \sim 1.6$ MPa.

Loading-unloading cycles tests in nanoindentation – for which the long relaxation time can be neglected at the time scale of the experiments – are well captured by a Kelvin-Voigt model as shown Supplementary Fig. 12e. The experimental data (represented with colored markers, using 10 bins per sample to average all data series) follow the model $\epsilon = (t/T)^{2/3}$, where $T = (2R^2 E_i \tau/(3\dot{F}_i))^{1/2}$, with a single value, $E_i\tau$ being fitted to the curves. To confirm the model, we perform FEM numerical simulations of indenting a bilayer composed of a thin elastic film of Young's modulus $E_f = 100$ MPa deposited onto a soft viscoelastic substrate ($E = 100$ kPa, $\tau = 10$ s) at different loading rates (10^{-1} to 10^{0} μN/s). Supplementary Fig. 12f shows a snapshot of the FEM simulation, the results of which, shown as dashed lines in Supplementary Fig. 12e, also follow the master curve.

10 *in vivo* pressure recording and artificial pressure increase

Supplementary Fig. 13 shows the temporal evolution of pressure P (black curve) and stretch λ (red) for four distinct flies. We observe that pressure initially increases, reaching a plateau of $\approx 1.5 - 3.5$ kPa during which most – at least 50% – of the wings deployment occurs.

We wonder whether wing deployment can be triggered by an external artificial increase of pressure. To test this hypothesis, a wild-type fly is placed under ether vapor for 10 minutes immediately after emerging from the pupal case. We then puncture its scutellum using a glass capillary (50-75 μm outside diameter) connected to a syringe pump and a pressure sensor. PBS is injected to impose different pressure plateaus and the fly is observed for at least 2 minutes to identify any sign of deployment before increasing the pressure. No wing deployment

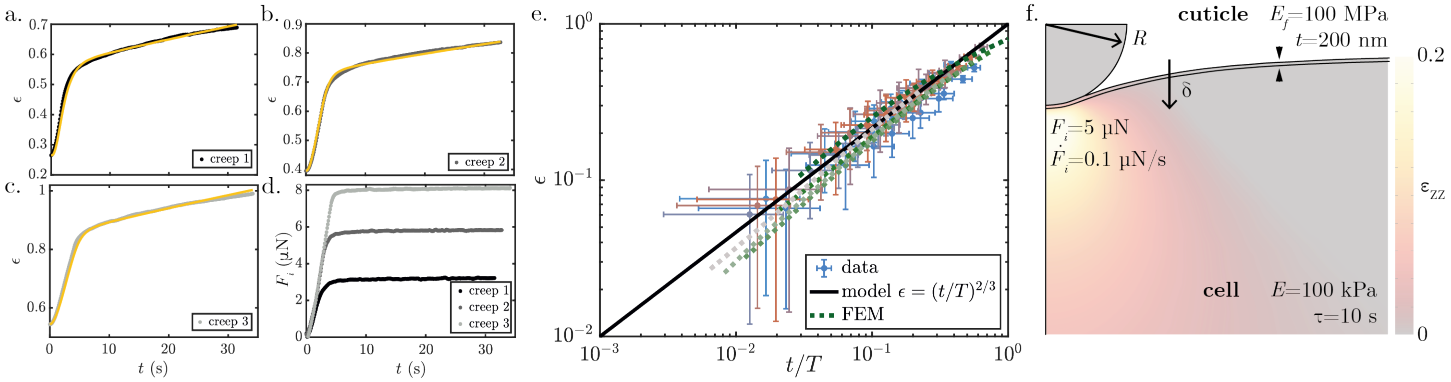

Supplementary Figure 12: **Nanoindentation on folded wings. (a-c)** Deformation $\epsilon = (\delta/R)^{1/2}$ as a function of time for three different creep experiments (experimental data: in black; Maxwell-Jeffrey fit: yellow line), each one undergoing a different force plateau F_i (see **(d)** for the corresponding loading signals). **(e)** ϵ versus normalized time t/T from nanoindentation experiments. Data (colored markers, using 10 bins per sample to average all experiments (n=6), standard deviation shown with errorbars) and FEM (dashed lines, from slow indentation $\dot{F}_i = 10^{-1}$ μN/s in dark green, to faster $\dot{F}_i = 10^{1}$ μN/s in gray) collapse on the model $\epsilon = (t/T)^{2/3}$ (black line). **(f)** FEM numerical simulation of the indentation of a bilayer composed of a visco-elastic cell substrate (Young's modulus $E = 100$ kPa, internal time $\tau = 10$ s) covered with an elastic cuticle film (thickness $t = 200$ nm, Young's modulus $E_f = 100$ MPa). This snapshot is taken as the indenter (radius $R = 4.7\,\mu$m) imposes a force $F_i = 5\,\mu$N at rate $\dot{F}_i = 10^{-1}$ μN/s, corresponding to the dark green curve in (b).

is observed below the pressure plateau of $P \sim 10$ kPa. Between $P = 10 - 16$ kPa, the wings deploy in about 10 minutes. Supplementary Fig. 14a and Supplementary Movie 10 show top- and side-view snapshots of such an experiment. The wings curved upwards. A physical reason for curvature may be a post-mortem difference in the elastic properties of the dorsal and ventral plates. Such asymmetry would result in curvature under pneumatic actuation, with the softer plates stretching more than the stiffer one, a property widely used in soft robotics to achieve curvature [8]. At higher pressure ($P > 17$ kPa), the pillars break, the ventral and dorsal layers delaminate, resulting in blistered or even balloon-like wings (see Supplementary Fig. 14b). Note that at this stage, increasing the pressure does not further stretches the tissue, which further justifies the use of a hyperelastic strain-stiffening model to describe the wing.

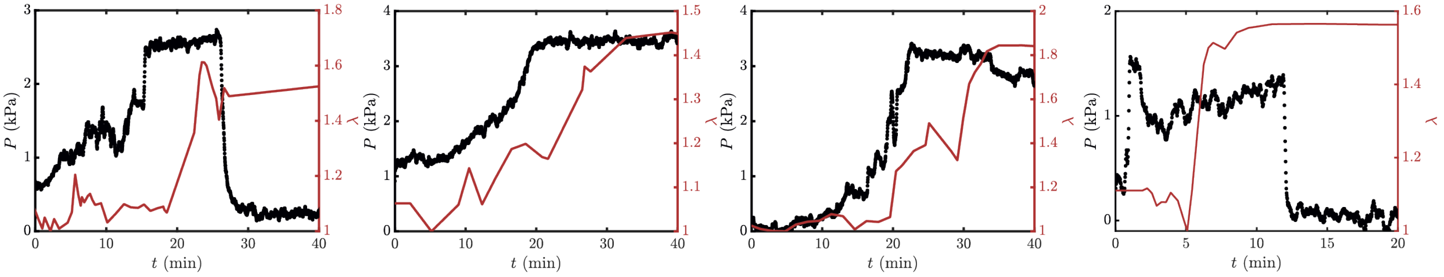

Supplementary Figure 13: ***in vivo* pressure measurements.** Pressure recording (black curves) in 4 different flies and corresponding stretch λ (red) in time as the wings deploy. Most of the wing deployment happens on a pressure plateau of $1.5 - 3.5\,$kPa.

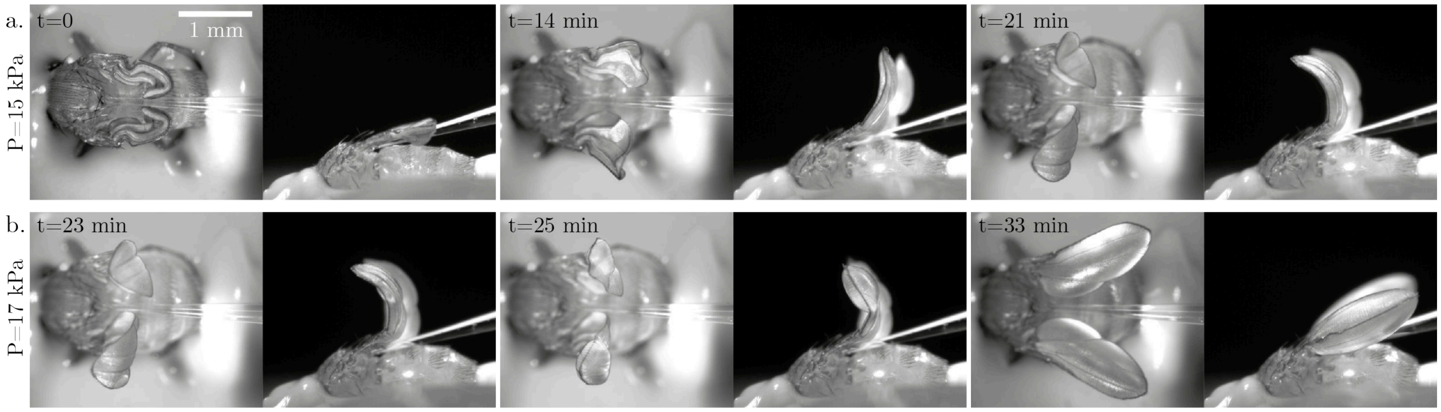

Supplementary Figure 14: **Artificial pressure increase. (a)** A pressure plateau of $P = 15$ kPa is applied in a fixed wild type fly with folded wings, triggering curved wings deployment in ~ 10 minutes. **(b)** An additional increase in the pressure to $P = 17$ kPa leads to microtubule pillars breakage and wing blade delamination resulting in blistered or ballon-like wings.

References

[1] Tsuboi, A., Fujimoto, K. & Kondo, T. Spatiotemporal remodeling of extracellular matrix orients epithelial sheet folding. *Sci. Adv.* **9**, eadh2154 (2023).

[2] Hurd, T. R., Liang, F.-X. & Lehmann, R. Curly encodes dual oxidase, which acts with heme peroxidase curly su to shape the adult drosophila wing. *PLoS genetics* **11**, e1005625 (2015).

[3] Lessinnes, T., Moulton, D. E. & Goriely, A. Morphoelastic rods part ii: growing birods. *Journal of the Mechanics and Physics of Solids* **100**, 147–196 (2017).

[4] Jones, G. W. & Chapman, S. J. Modeling growth in biological materials. *Siam review* **54**, 52–118 (2012).

[5] Andersen, S. O. Insect cuticular sclerotization: a review. *Insect biochemistry and molecular biology* **40**, 166–178 (2010).

[6] Wagner, R., Pittendrigh, B. R. & Raman, A. Local elasticity and adhesion of nanostructures on drosophila melanogaster wing membrane studied using atomic force microscopy. *Applied surface science* **259**, 225–230 (2012).

[7] Siéfert, E. & Roman, B. Morphogenesis through elastic phase separation in a pneumatic surface. *C. R. Mec.* **348**, 649–657 (2020).

[8] Ilievski, F., Mazzeo, A. D., Shepherd, R. F., Chen, X. & Whitesides, G. M. Soft robotics for chemists. *Angewandte Chemie International Editiion* (2011).
