# Supplementary material for: Mechanics of Drosophila wing deployment
Source: Nat Commun. 2024 Dec 11;15:10577. doi: 10.1038/s41467-024-54527-0 (PMC11634967; doi:10.1038/s41467-024-54527-0)
Supplement: Supplementary file 2 — Description of Additional Supplementary Information [file 41467_2024_54527_MOESM2_ESM.docx]

**Description of Additional Supplementary Files**

File Name: Supplementary Movie 1

Description: Wing deployment (wild type female Drosophila) from top and side views (timer mm:ss).

File Name: Supplementary Movie 2

Description: Side view of a fly increasing its internal pressure by swallowing air (white arrow indicates pharyngeal pumping) and contracting abdominal muscles.

File Name: Supplementary Movie 3

Description: Micro-CT scan of a folded wing (ImageJ plugin 3D viewer). Z-stack of cross sections of the same wing normal to the proximo-distal axis reveals the internal structure (i.e. dorsal and ventral plates connected by pillars).

File Name: Supplementary Movie 4

Description: Binocular fluorescent microscopy of hemolymph flows labeled with fluorescent beads during deployment (left) and in an adult wing (right). Hemolymph invades the entire structure during deployment, in stark contrast to hemolymph flow in an adult wing, where it is restricted to the network of veins.

File Name: Supplementary Movie 5

Description: *in vivo* measurement of internal pressure during wing deployment. The scutellum of a newly emerged fly is poked with a glass capillary connected to a pressure sensor, and wing deployment is recorded. Inset: measured pressure P(*t*) over time.

File Name: Supplementary Movie 6

Description: Combining recordings of wing deployment in 6 individuals shows reproducibility of the process (3 females above, 3 males below). The time at which the wings begin to deploy is chosen as the reference for combining the videos.

File Name: Supplementary Movie 7

Description: 3D reconstruction of the distal tip of a wing during deployment obtained with a two-photon microscope, enabling a measurement of the wing thickness in vivo of ~ 35 µm during deployment (while the thickness is ~ 18 µm before deployment, see TEM Fig.3b and quantification boxplot “wing thickness" Fig.3e).

File Name: Supplementary Movie 8

Description: 3D reconstruction of the apical surface of a single cell (Utrophin:GFP) from a z-stack obtained by fluorescent spinning disk microscopy. Integration of the 3D shape enables measurement of the apical cell surface area (see Folded condition of the boxplot “3D Area", Fig.3e).

File Name: Supplementary Movie 9

Description: Tensile test on a dissected folded wing. The distal end of the wing is connected to a force sensor, while we impose a displacement on the proximal end glued to a moving stage (see Fig.4b and Supplementary Fig.8c for the measured stress-strain curves).

File Name: Supplementary Movie 10

Description: An artificial increase in pressure triggers wing deployment. We poke the scutellum of a sacrificed, newly emerged wild type fly with a glass capillary connected to a syringe. We impose a first pressure plateau of 15 kPa, at which point the wings unfold and curl upward. At t=23 minutes a further increase in the pressure plateau to 17 kPa leads to rupture of the microtubule pillars and delamination of the dorsal and ventral layers, resulting in balloon-like wings (see Supplementary Fig.14 for corresponding snapshots).

File Name: Supplementary Movie 11

Description: 3D FEM simulations (COMSOL Multiphysics) of the inflation of wing-like structure composed of two plates of thickness e=6.5 µm connected by pillars (height h=7.5 µm, diameter d=3.3 µm, interpillar distance a=6.2 µm). The visco-hyperlastic material (E=100 kPa, 𝐽 =20, =10 s) is 𝑚 τ submitted to a pressure step of P=16 kPa at t=0. Color indicates in-plain strain.

File Name: Supplementary Movie 12

Description: Wing deployment in a Curly mutant from a top- and side-view. Scale bar: 1 mm; timer mm:ss.

File Name: Supplementary Movie 13
Description: Elastic recovery after the interruption of wing deployment in a wild-type fly. Scale bar: 1 mm; timer mm:ss.
